# Space distribution of EEG responses to hanoi-moving visual and auditory stimulation with Fourier Independent Component Analysis

**DOI:** 10.3389/fnhum.2015.00405

**Published:** 2015-07-21

**Authors:** Shijun Li, Yi Wang, Guangyu Bin, Xiaoshan Huang, Dan Zhang, Gang Liu, Yanwei Lv, Xiaorong Gao, Shangkai Gao, Lin Ma

**Affiliations:** ^1^Department of Medical Instruments, PLA General HospitalBeijing, China; ^2^Department of Stomatology, PLA General HospitalBeijing, China; ^3^Department of Biomedical Engineering, School of Medicine, Tsinghua UniversityBeijing, China; ^4^Department of Radiology, PLA General HospitalBeijing, China; ^5^Clinical Epidemiology and Biostatistics Research Office, Beijing Research Institute of Traumatology and OrthopaedicsBeijing, China

**Keywords:** electroencephalography, action-related visual and auditory stimulation, Fourier-Independent Component Analysis, sensory-motor area, source signals

## Abstract

**Background and objective:** The relationship between EEG source signals and action-related visual and auditory stimulation is still not well-understood. The objective of this study was to identify EEG source signals and their associated action-related visual and auditory responses, especially independent components of EEG.

**Methods:** A hand-moving-Hanoi video paradigm was used to study neural correlates of the action-related visual and auditory information processing determined by mu rhythm (8–12 Hz) in 16 healthy young subjects. Independent component analysis (ICA) was applied to identify separate EEG sources, and further computed in the frequency domain by applying-Fourier transform ICA (F-ICA).

**Results:** F-ICA found more sensory stimuli-related independent components located within the sensorimotor region than ICA did. The total number of independent components of interest from F-ICA was 768, twice that of 384 from traditional time-domain ICA (*p* < 0.05). In the sensory-motor region C3 or C4, the total source signals intensity distribution values from all 14 subjects was 23.00 (Mean 1.64 ± 1.17) from F-ICA; which was more than the 10.5 (Mean 0.75 ± 0.62) from traditional time-domain ICA (*p* < 0.05). Furthermore, the intensity distribution of source signals in the C3 or C4 region was statistically significant between the ICA and F-ICA groups (strong 50 vs. 92%; weak 50 vs. 8% retrospectively; *p* < 0.05). In the Pz region, the total source signal intensity distribution from F-ICA was 12.50 (Mean 0.89 ± 0.53); although exceeding that of traditional time-domain ICA 8.20 (Mean 0.59 ± 0.48), the difference was not statistically significant (*p* > 0.05).

**Conclusions:** These results support the hypothesis that mu rhythm was sensitive to detection of the cognitive expression, which could be reflected by the function in the parietal lobe sensory-motor region. The results of this study could potentially be applied into early diagnosis for those with visual and hearing impairments in the near future.

## Introduction

Human auditory and visual perception can promote the simultaneous activation of motor regions responsible for sensory relevant actions (Rizzolatti et al., 1996; Gallese and Goldman, 1998; Iacoboni et al., 2005). Such motor-auditory-visual integration ability is of great significance for the formation of a variety of cognitive functions, including language, memory etc. Efficient integration is supported by the highly interconnected neural network across the sensory processing regions and the motor controlling region (Rizzolatti and Craighero, 2004).

The neural correlates for the motor-auditory-visual integration have been first reported in animal studies showing that after observing people grabbing food with their hands, the neurons in the sensory-motor cortex of Macaque monkeys exhibited activity patterns associated with hand-grabbing action (Rizzolatti et al., 1996; Gallese and Goldman, 1998). Similarly, action-related sounds have been shown to trigger action specific neuronal activities in the monkey premotor cortex without the execution of the actions (Kohler et al., 2002). Furthermore, it has been reported that dysfunction of the neurons at critical sensory-motor integration regions, i.e., Convergence–Divergence Zones (CDZs), is closely associated with diseases of action imitation barriers, visual understanding obstacles, auditory comprehension obstacles, language defects, motor defects and Autism Spectrum Disorder (ASD; Iacoboni et al., 2005; Moseley et al., 2008; Rizzolatti and Sinigaglia, 2008; Le Bel et al., 2009; Neuper et al., 2009). Therefore, identification of the neural correlates involved in this integration process is potentially useful for the clinical diagnosis of those cognitive dysfunctions.

One approach to investigating the neural correlates is to perform an electroencephalography (EEG) and to measure the Mu Rhythms (8–12 Hz) brain activity over the sensorimotor cortex. The decrease of the mu rhythm power is believed to reflect an increase of the neuronal activity for action preparation or execution (Pfurtscheller and Lopes da Silva, 1999). Recent studies in humans have suggested that the inhibition of the mu rhythm activity (power decrease) may reflect the function of connecting action, understanding information, processing responses, and being able to transfer “see” and “hear” into “action” (Pineda, 2005; Alegre et al., 2010; Heida et al., 2014). Previous studies from us and others also demonstrated that action-related auditory stimuli led to event-related spectral perturbation in the mu rhythm (i.e., 8–12 Hz) over the motor cortex (Li, 1998; Bangert and Altenmüller, 2003; Neuper et al., 2006). However, the question of how much of the EEG activity originated from the stimulation tasks and how many of the source signals associated with these EEG activities was not well-addressed in those studies. Therefore, a data analysis method with sufficient sensitivity and reliability is necessary to detect the mu rhythm based neural correlates of the action-related visual and auditory information processing.

In this study, we aimed to use a modified independent component analysis (ICA) method to capture the neural correlates for motor-auditory-visual integration. ICA is a computational method for separating a multivariate signal into additive subcomponents with the assumption that the subcomponents are non-Gaussian signals and that they are statistically independent from each other (Comon, 1994). ICA method was applied to solve the unknown source signal's space distribution by separating the independent components from the hypothetical source signals of the known EEG signals (Vigario et al., 2000; Hyvärinen et al., 2010). Makeig et al. first applied ICA into EEG signal processing and conducted an experiment in which the subject was instructed to press a button at the prompt of auditory stimulus: 14 components were extracted, with the θ wave being prominent in component 1, α wave in component 2, and the low-frequency eye movement in another different independent component (Makeig et al., 1996). Compared with Principal Component Analysis (PCA), ICA was more applicable for extracting spatially distinct EEG components (Makeig et al., 1997). To date, ICA has been widely applied in neuroimaging data processing such as EEG and fMRI, for the extraction of task-specific brain responses, signal artifacts, etc. (e.g., Vigario, 1997; Jung et al., 1999; Joyce et al., 2004; Beckmann et al., 2005). However, the classical ICA method may not be applicable for extracting rhythmic EEG activities, as the non-Gaussian assumption held by the ICA algorithm is not compatible with the Gaussian nature of the rhythmic brain activities (Hyvärinen et al., 2010).

The Fourier-Independent Component Analysis (F-ICA) algorithm, a recently proposed revision of the ICA algorithm, allows the application of ICA for analyzing rhythmic brain activities (Hyvärinen et al., 2010). The F-ICA method is based on short-time Fourier transforms of the EEG signals, for the purpose of exploring the spatiotemporal and spectral patterns in the cortical source space over time scales of minutes (Hyvärinen et al., 2010; Ramkumar et al., 2012). Therefore, in this study, we will employ the F-ICA algorithm to analyze the EEG data generated when subjects received auditory or visual stimuli associated with a hanoi-moving task, which is performed by moving disks stacked on a peg to an adjacent peg. We used a complex mixing matrix in F-ICA to model different phases of the spatial extension source and fulfilled data analysis to search the source spatial distribution of the interested area. We expected the effective independent component to be found under the action-related visual and auditory stimulation phantom with F-ICA.

## Materials and methods

### Subjects

Sixteen healthy right-handed subjects (10 females, 6 males; ages: 18–22 years) participated in this study after giving written informed consent. Two subjects were excluded the study due to incomplete data recording. Handedness was assessed using the Edinburgh test (Oldfield, 1971). The study was approved by the Institutional Review Board of School of Medicine, Tsinghua University.

### EEG paradigm

Each participant was instructed to watch a recorded action video of one hand moving Tower of Hanoi (size: 720^*^720, speed: 1536 kpbs, length 8 s). Tower of Hanoi (TOH) consisted of three pegs, and four colored disks stacked on one of the pegs (Wright and Hardie, 2015). The 8 s-long video experiment consisted of a 5 s period of one hand moving one disk from one peg to another, followed by a 3 s resting period. Sound was produced by one hand moving the disk from the peg at the beginning of 0s, as the auditory stimuli. The video playing was divided into three phases: (1) Pre-learning: the subjects repeatedly listened to the video synchronous sound, but did not watch the video images. (2) Learning: the subjects simultaneously watched the video images and listened to the synchronous sound. The subjects knew which action produced the sound, so as to establish an association of the sound and the action. (3) Post-learning: the subjects repeatedly listened to the video synchronous sound, but did not watch the video images. Each phase consisted of 4 video playing sessions; in each session, the 8 s-long experiment was performed 15 times. There was a 4 s interval rest between each video experiment and a 120 s interval rest between each two-consecutive sessions. A total of 60 (4×15) video experiments of 8 s EEG data were collected in each phase. During the experiments, the subjects were instructed to count the number of times they heard the sounds. At the end of each experiment, the subjects were asked which types of the sounds they heard, and how many times. This was to ensure that the subjects were focusing on the experiments during the testing.

### EEG data recording

A Neuroscan EEG machine was used to acquire data from 32 electrodes channels according to the International 10–20 System of Electrode Placement. The data acquisition rate was 200 Hz. First, a 4–40 Hz bandwidth filtering was applied to the data. Then, a total of 240 (60×4) s (1–5 s) of EEG data from the “Learning” phase was selected as the task status. Lastly, the other two phases (pre-learning and post-learning) data were combined, forming the data matrix with a size of 30×96,000; 30 = electrode numbers; 96,000 = 2 (phases) ×240 s ×200 Hz.

### Algorithm

Traditional time-domain ICA (T-ICA) was used to automatically extract and remove eye movement artifacts, according to previous studies (Bingham and Hyvärinen, 2000; Joyce et al., 2004), and then Fourier-ICA (F-ICA) was performed from time-domain ICA analysis (Hyvärinen et al., 2010; Ramkumar et al., 2012). We adopted previous F-ICA algorithm methods to extract EEG rhythmic activity signals from EEG data. The process included (1) adopting random low frequency signal to modulate 10 Hz sine signal, (2) obtaining amplitude modulation sine signal, and (3) analyzing the probability density distribution of the signal amplitude. The distribution of amplitude modulation sine signal has two properties. First the amplitude distribution of pure sine signal is bimodal with strong negative kurtosis. Therefore, it has strong non-Gaussian distribution. Secondly, the effectively normal distribution can be computed when a smaller amplitude modulation is added, thus reinforcing the Gaussian distribution of the time-domain amplitude and its probability density signal (Hyvärinen et al., 2010).

ICA was described as a blind source separation method (Wang et al., 2008). It assumes that the observation signal *x*(*t*) = [*x*_1_(*t*), …, *x_m_* (*t*)]^*T*^ to be a set of multi-channel random signals and obtained from a set of independent source signals and a linear transformation mixed matrix A of *s*(*t*) = [*s*_1_(*t*), …, *s_n_* (*t*)]^*T*^(1).

(1) $$\begin{array}{r} x\left(t\right)=As\left(t\right) \end{array}$$

Generally, it was assumed that the number of source signals was less than or equal to the channel number of the observation signals. In the model, the source signals (*t*) and the mixing matrix A are unknown. Only after “m” time was processed could the mixing be observed. ICA is to solve unmixing matrix W under the condition of the unknown mixing matrix A and source signal s(*t*). Therefore, the solved signal *u*(*t*) = [*u*_1_(*t*), …, *u_n_* (*t*)]^*T*^ is to be approximate to the source signal s(*t*). The relationship ***u***(*t*), ***s***(*t*) and ***x***(*t*) is expressed as Equation (2).

(2) $$\begin{array}{r} u\left(t\right)=Wx\left(t\right)=WAs\left(t\right) \end{array}$$

When the information transmission reached the maximization, the mutual information between the output terminal components reached the minimum (Bell and Sejnowski, 1995). So, the W enables the objective function to reach maximum and to obtain the ICA solution.

The F-ICA model was assumed to be a linear, instantaneous hybrid model similar to ICA. In the model, EEG measurement data were expressed as *x*_*c*,τ_, in which c is EEG electrode number, and τ istime sequence. Each EEG signal was assumed to be linear aliasing of the source signal *s*_*p*,τ_(3), where p is each source number. In the equation below *a*_*c*,*p*_ is an aliasing coefficient. All sources are statistically independent and randomly processed.

(3) $$\begin{array}{r} x_{c,\tau }=\overset{p}{\underset{p=1}{\sum }}a_{c,p}s_{p,\tau } \end{array}$$

The basic idea of F-ICA is to transform the signals to the frequency domain by Fourier transformation, and then to use ICA to decompose data. First the measurement data were divided into a series of assumed time bucket signal T, each time bucket containing N sample point data. The measurement data *x*_*c*,τ_ can be decomposed into a series of time bucket data (Equation 4).

(4) $$\begin{array}{r} {}[\left(x_{c,1},\cdots \;,x_{c,N}\right),\left(x_{c,N+1},\cdots \;,x_{c,2N}\right),\cdots \;,\\ \left(x_{c,\left(T-1\right)N+1},\cdots \;,x_{c,TN}\right)] \end{array}$$

Fourier transformation was carried out on these sets of time bucket data respectively to obtain sets of frequency domain data $\hat{x}$_*c,tf*_, where *f* = 1,2, …, F. F is the index of Fourier coefficient. The Fourier frequency domain coefficients were joined together to form a two-dimensional data $\hat{x}$_*c,tf*_. By Fourier transform nature, the matrix after transformation can satisfy Equation (5).

(5) $$\begin{array}{r} {\hat{x}}_{c,tf}=\overset{p}{\underset{p=1}{\sum }}a_{c,p}{ŝ}_{p,tf} \end{array}$$

Equation (5) is also a typical ICA model as Equation (1). Whereas the source signal $\hat{s}$_*p,tf*_ and measurement signal $\hat{x}$_*c,tf*_ are plural, only *a*_*c*,*p*_ is a real number. Adopting Complex-valued ICA to decompose two-dimensional matrix $\hat{x}$_*c,tf*_ can estimate the mixing coefficient *a*_*c*,*p*_ as well as unmixing matrix W.

Through comparing various objective functions, we adopted robust measurement function in F-ICA as the target function (Equation 6), which had better separate rhythmic source signal (Hyvärinen et al., 2010). In the objective function, due to the introduction of the logarithmic function, zero crossing value will not rise too fast, nor be sensitive to the boundary. Its stability is higher than that of the traditional fourth order statistics (namely Kurtosis) method.

(6) $$\begin{array}{r} g_{4}\left(y\right)=\text{log}\left(eps+y\right) \end{array}$$

Where *eps* is constant, *g*(·) is the non-linear function and *g*_4_(·) is the robust measure function. After using the robust measure function as a non-linear function, and assuming $\hat{s}$_*p*_ is normalized to zero, mean and unit step variable, the final optimization goal *J*($\hat{s}$_*p*_) can be expressed as Equation (7).

(7) $$\begin{array}{r} J\left({ŝ}_{p}\right)=\frac{1}{TF}\underset{tf}{\sum }-\log\left(1+{\left|{ŝ}_{p,tf}\right|}^{2}\right) \end{array}$$

### Simulation

It has been known from the previous research studies that auditory stimulus during post-learning phase can cause α (8–13 Hz) rhythmic desynchronization phenomenon at motor cortex region (Pineda, 2005; Li et al., 2011). In addition, it was shown that mu (8–12 Hz) rhythmic desynchronization in sensory-motor area may be accompanied by β (14–25 Hz) rhythmic desynchronization phenomenon (Pineda, 2005). Therefore, the simulation data in this paper comprised three EEG rhythmic components and three noise components to minimize the desynchronization phenomenon. The first source component was 10 Hz amplitude modulation signal which simulates the mu rhythm component near C3/C4 electrode. The second source component was 10 Hz amplitude modulation signal, which simulates α rhythmcomponent near Pz electrode. The third source component was 20 Hz amplitude modulation signal, which simulates β rhythm component of the motor cortex region. The first noise component S4 was a single spike. The second noise component S5 was a spike for multiple locations. The third noise component S6 was a wide spectrum white noise, which simulates Electromyogram interference in EEG. The noise location of these simulation source signals or the moment of amplitude modulation signals could be randomly changed. The data length of the signal was 100 s and the sampling rate was 100 Hz (10000 points). After the source signal amplitude normalization, a 6×6 hybrid matrix was generated and six channels of data were processed.

F-ICA processing steps were as follows: (1) Data were divided into 100 segments with 1 s window or 100 points per 1 s. (2) 5–30 Hz bandwidth filter was applied on the data. (3) All data segments were joined into a two-dimensional matrix. (4) The mean value of the row was subtracted from each set of row data in the two-dimensional matrix. (5) ICA decomposition was performed by using the complex value ICA algorithm, and adopting Formula (Equation 7) for objective function; then the decomposed source signals and unmixing matrix were obtained.

The Infomax method criterion were used for data processing (Makeig et al., 1997), which applied the correlation of the unmixing matrix *W* and mixing matrix *A* to design the evaluation criterion. Ideally, the product of the unmixing matrix and the mixing matrix *WA* should be a permutation matrix, meaning that each row had a non-zero element with only 1 or –1. Therefore, the maximum of the absolute value of each row of the product matrix represented the estimation accuracy of the mixing coefficient of the corresponding source component. If the value was greater than 0.95, that meant the source component was correctly estimated. Computer simulation technology was used to simulate 600 multiple sets of source signals in six groups for ICA decomposition. The results were analyzed for statistical significance. If all of the source signals were separated correctly, a total of 300 rhythmic signal components and 300 noise components were correctly estimated.

Due to the statistical separation accuracy of F-ICA, 77% of the EEG rhythmic components and 35% of the noise components were correctly estimated. While in the traditional time-domain ICA method, only 20% of the EEG rhythmic components and 87% of the noise components were correctly estimated. The simulation results showed that F-ICA had a greater advantage in the separation of amplitude modulation signals. The brain rhythmic activity was similar to the amplitude modulation sine signal. Therefore, F-ICA may obtain better results in the separation of EEG rhythm components.

### Data preprocessing

The runica function of EEG was analyzed using software EEGLab which uses Infomax method to fulfill F-ICA decomposition. Non-linear function of tangent function was used to obtain 30 independent components of spatial patterns and spectrum. We adopted matrix A to represent the mixing coefficient. Each line of the mixing matrix A represented the intensity distribution of the corresponding source component in the measuring lead. Therefore, the unmixing matrix *W* and inverse matrix *W*^*T*^ represented the intensity distribution of the estimated source component in various measuring leads. A brain topographic map was used to compute the intensity distribution of each independent component (IC) at various leads, which can be used as the spatial pattern of the ICA to help finding the source of the interest brain signals.

### Data analysis

Data with normal distribution were described by mean and standard deviation and tested by paired-t test. Data with skewness distribution were described by median and quartile and tested by Wilcoxon rank test. The statistical software SPSS 18.0 (SPSS Inc., Chicago, IL) was used for the statistical analysis. *p* value < 0.05 was considered as statistically significant.

## Results

### EEG responses to hanoi-moving visual and auditory stimulation with time-domain ICA

**Figure 1:**
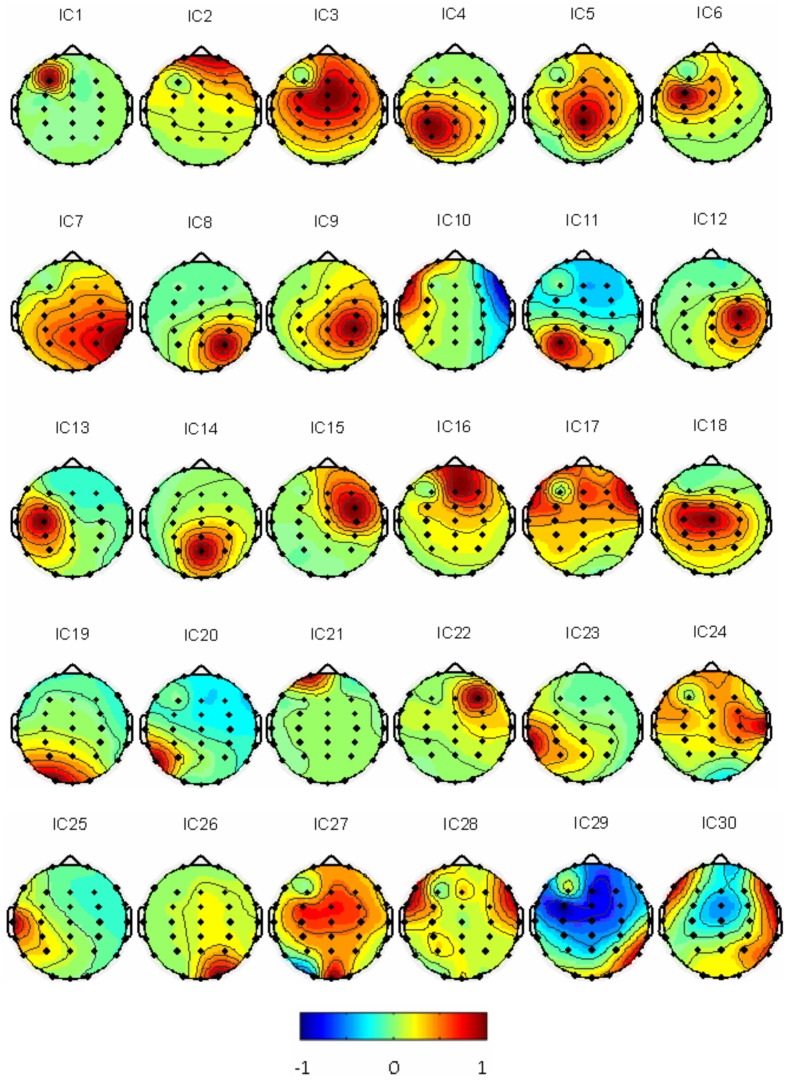
**Thirty IC spatial distributions in a representative subject (blue represents –1, red represents 1)**.

**Figure 2:**
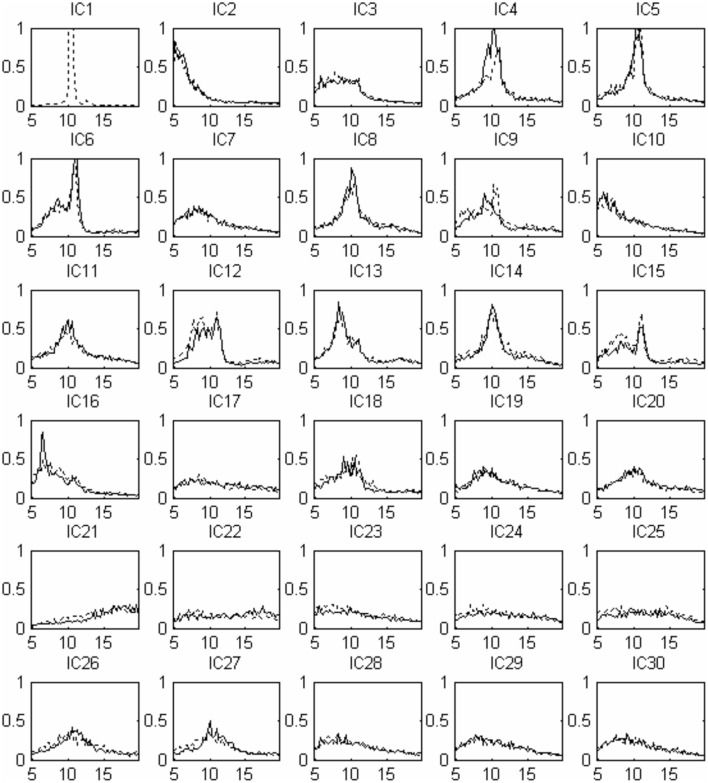
**Source signal power spectra curve corresponding to Figure 1: x-coordinate for frequency (Hz), y-coordinate for power spectrum (***u***V^2^)**. The solid line indicates pre-learning, the dotted line indicates post-learning.

**Figure 3:**
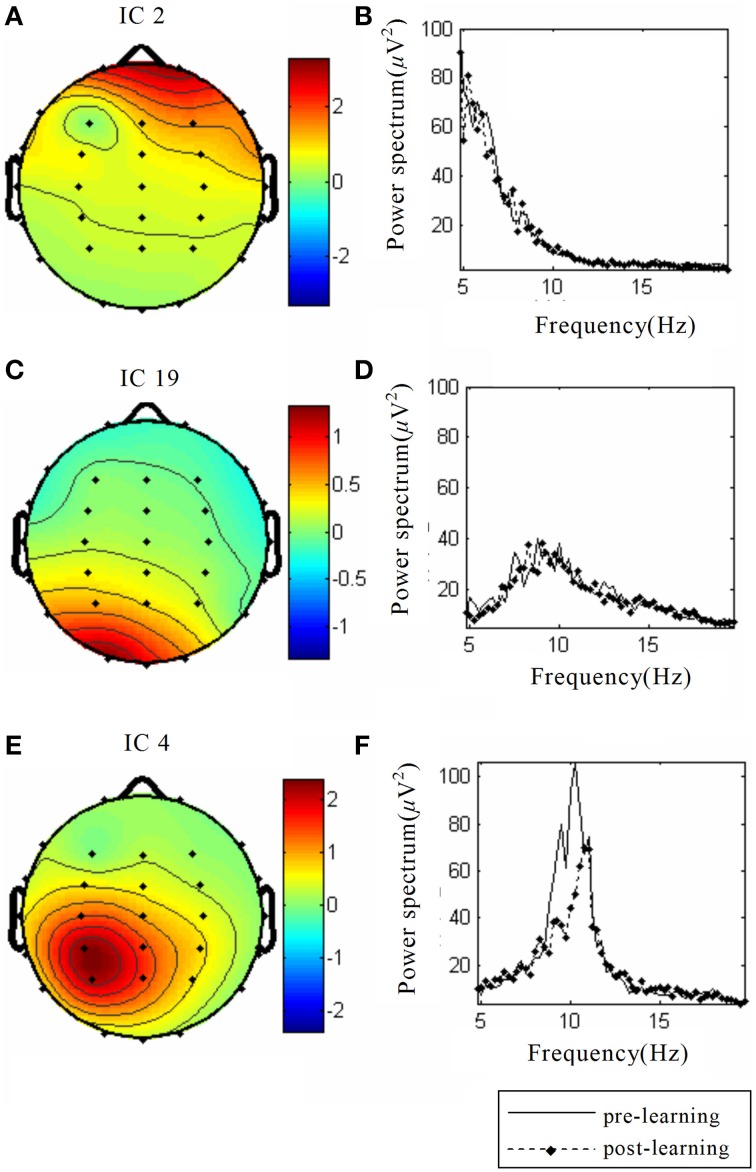
**The spatial distribution and the corresponding power spectrum diagram of three interested source signals**. The source data come from simulation, including **(A)** Eye power signal IC2, **(C)** α rhythm source activity IC19, **(E)** Auditory stimuli evoked brain source activity IC4; **(B)**, **(D),** and **(F)** Power spectra of the above three source signals, respectively. The solid line indicates the pre-learning, and the dotted line indicates post-learning.

Thirty independent components of spatial patterns from one of the subjects are shown in Figure 1 with time-domain ICA (T-ICA) analysis method. The spatial distribution of source signals is denoted by color circles and the signal intensity (–1 to 1) by the color on the map. Corresponding power spectrum from each independent component is displayed in Figure 2. The results in Figure 2 also show that the mu rhythm's power at the motor cortex region was lower at post-learning phase than at pre-learning (IC4). According to the spatial pattern brain topographic map and the power spectra of each IC at pre-learning and post-learning phases, three typical IC components were identified: IC2, IC19, and IC4. The details of the interested source signals are as follows:

1. IC2: (i) Components were mainly distributed in frontal region (Figure 3A); (ii) Power spectra were mainly low frequency signals (Figure 3B).This component was electro-oculogram.
2. IC19: (i) Components were mainly distributed in occipital region (Figure 3C); (ii) Power spectra were mainly distributed in α band of 8–12 Hz (Figure 3D); (iii) there was no difference in the power spectra between the components of pre-learning and post-learning phases. So, IC19 represented α activity of the occipital region.
3. IC4: (i) Components were mainly distributed in the motor region and top region of the left hemisphere (Figure 3E); (ii) Power spectra were mainly located in 8–12 Hz, and in the same frequency range of mu rhythm (Figure 3F); (iii) 8–12 Hz rhythm energy during the post-learning task was lower than during pre-learning task, suggesting that IC4 brain activation at post-learning was higher than at pre-learning.

**Figure 4:**
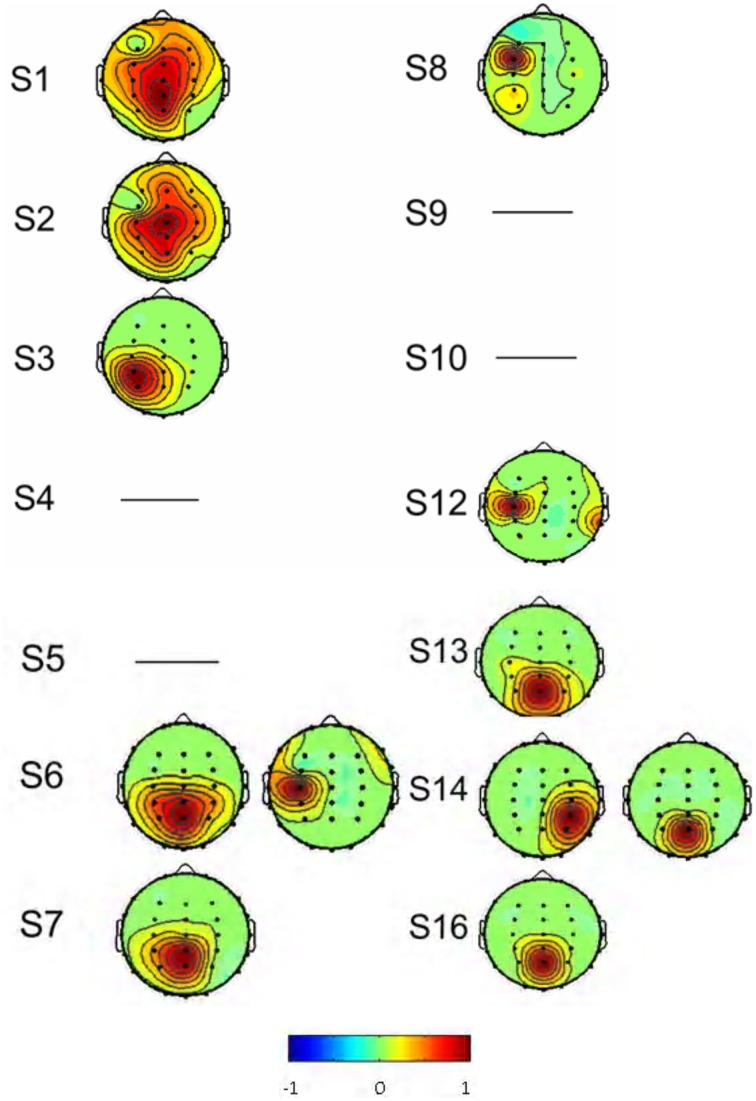
**The spatial distribution of the interested source signals from ICA analysis**. Results were from 14 of the 16 subjects, and subjects S 11 and S 15 were excluded. No right source components were found in subjects S4, S5, S9, and S10 and the rest subjects had normal source distributions. Signal space distribution was denoted by selected color circles and color intensity (–1 to 1) on the map. Blue represents –1, Red represents 1.

As described in the “Methods” above, the decomposed 384 sources of the 14 subjects were manually selected (Table 1). Eight subjects (S1, S2, S3, S7, S8, S12, S13, and S16) had one interested IC (Figure 4) and two subjects (S6, S14) had two interested ICs. Four subjects (S4, S5, S9, and S10) had no suitable source selected. The spatial distribution of source signals of the 14 subjects is also displayed in Figure 4. The signal space distribution is denoted by selected color circles and by the intensity of the color (–1 to 1) on the map in each IC region. Each circle was given an intensity distribution value from 0 to 1 based on the intensity distribution of the corresponding source component in the measuring lead as described in methods. The source signal distributions in the Pz and C3/C4 regions are also depicted in Table 1; where the source signals was expressed as the sum of intensity distribution values for an IC in the a region. Figure 4 and Table 1 show that while many subjects had a source component near Pz region, five subjects (S3, S7, S8, S13, S16) had weak source signals (intensity distribution value < 1.0) located in the primary motor cortex region near C3 or C4; and five (S1, S2, S6, S12, S14) had strong source signals (value ≥1.0) in the region (Figure 4 and Table 1).

**Table 1:** **Number of source signals and sum of intensity distribution (ID) values in Pz and C3/C4 between T-ICA and F-ICA group**.

**No**.	Source signals^*^		**T-ICA (ID)**		**F-ICA (ID)**	
	**T-ICA (#IC)**	**F-ICA (#IC)**	**Pz**	**C3/C4**	**Pz**	**C3/C4**
S1	32 (1)	32 (1)	1.00	1.50	0.25	1.50
S2	32 (1)	32 (1)	1.00	1.50	1.00	0.50
S3	32 (1)	96 (3)	0.50	0.75	1.50	3.50
S4	0 (0)	64 (2)	0.00	0.00	0.75	1.00
S5	0 (0)	64 (2)	0.00	0.00	1.25	1.50
S6	64 (2)	64 (2)	1.20	1.75	1.50	1.75
S7	32 (1)	64 (2)	1.00	0.75	1.00	2.50
S8	32 (1)	64 (2)	0.25	0.50	1.00	1.50
S9	0 (0)	64 (2)	0.00	0.00	0.75	2.00
S10	0 (0)	0 (0)	0.00	0.00	0.00	0.00
S12	32 (1)	0 (0)	0.25	1.25	0.00	0.00
S13	32 (1)	64 (2)	1.00	0.75	1.00	1.00
S14	64 (2)	96 (3)	1.00	1.25	1.75	4.00
S16	32 (1)	64 (2)	1.00	0.50	0.75	2.25
Total	384 (12)	768 (24)	8.20	10.50	12.50	23.00
Avg			0.59 ± 0.48	0.75 ± 0.62	0.89 ± 0.53	1.64 ± 1.17

* *Number of source signals was defined as the number of electrodes 32 times the number of Independent Components (#IC; Figures 4, 7) found for each patient. The number of source signals from all 14 patients was added to obtain the total source signals. ID: Spatial distribution of source signals is denoted by color circles and color intensity on the map (Figures 4, 7). Each circle was given an intensity distribution (ID) value from 0 to 1 based on the calculation described in “Methods.” The above ID value was the sum of the values in parietal-occipital area (Pz) and sensorimotor area (C3 and C4), respectively. Sum of ID value: 0, no signal; < 1.0, weak signals; ≥1.0, strong source signals found. Avg = Mean ± SD*.

### EEG responses to hanoi-moving visual and auditory stimulation with fourier-ICA

**Figure 5:**
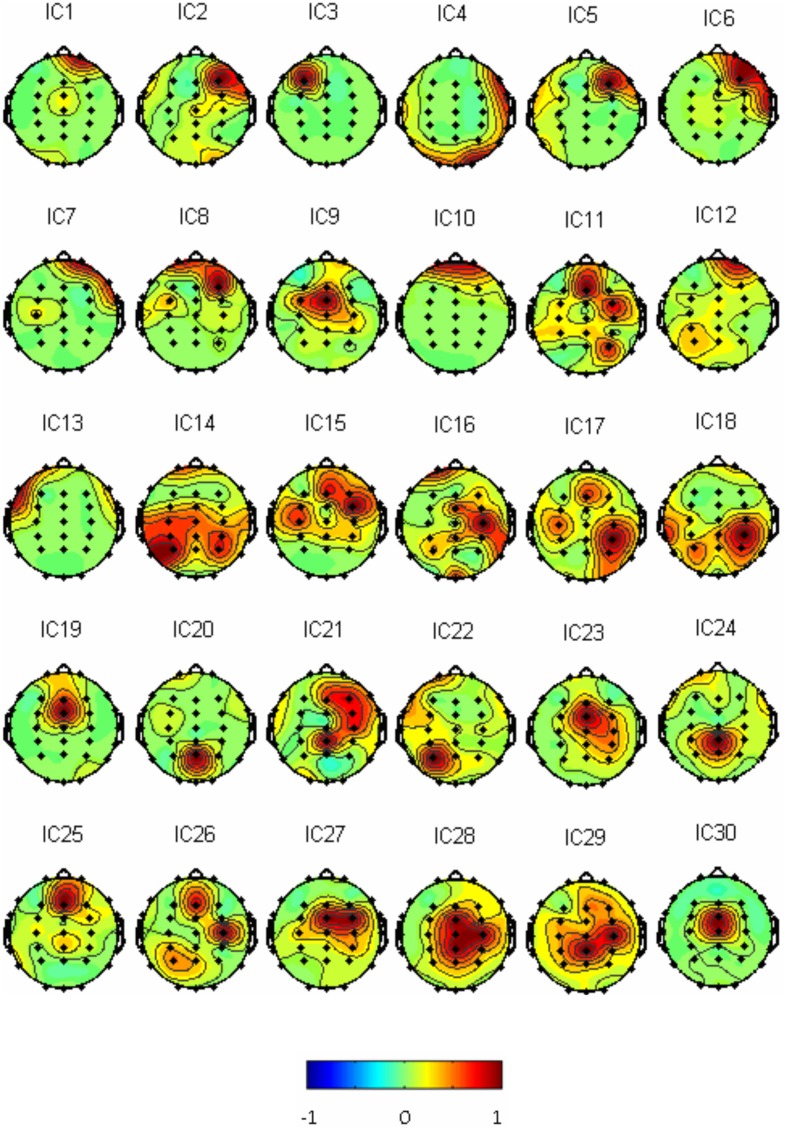
**Thirty Fourier ICA spatial distributions of the subject S3 (blue represents –1, red represents 1)**.

**Figure 6:**
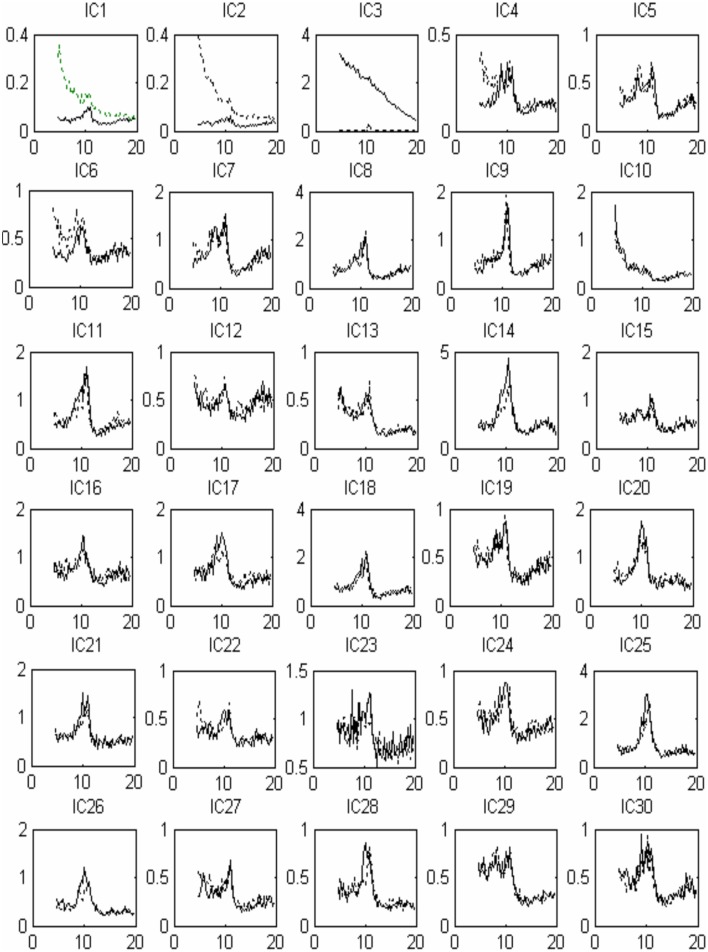
**Source signal power spectra curve corresponding to Figure 5: x-coordinate for frequenc y (Hz), y-coordinate for power spectrum (***u***V^2^)**. The solid line indicates pre-learning, and the dotted line indicates post-learning.

As described in the “Methods” for F-ICA processing steps above, IC power spectrum was used to select the decomposed 768 source signals from the 14 subjects (Table 1). Figure 5 shows 30 ICs analysis results of subject S3 and Figure 6 shows the corresponding power spectra curve of 30 ICs in Figure 5. The results of the interested source signals are as follows:

1. IC10 was mainly distributed in the region of the forehead, and the power spectra were mainly low frequency signals. The component was electro-oculogram; similar to the IC2 analysis results displayed in the time-domain ICA above (Figure 1).
2. IC14 was mainly located in the parietal region. IC20 was located in occipital top region near Pz. IC26 was located in the primary motor cortex region on the right side. The power spectra curve showed that all these three components had a significant difference between the two task phases; in which the mu rhythm power spectra values were higher during pre-learning task than that during post-learning task (Figure 5). These results indicated that the three components represented the activities related with tasks.
3. IC17, IC18 and IC22 were located in the occipital region. The power spectra were mainly distributed in α band of 8-12 Hz. There was no difference in the power spectra of the components between pre and post-learning. The three components represented α activity in the occipital region.

**Figure 7:**
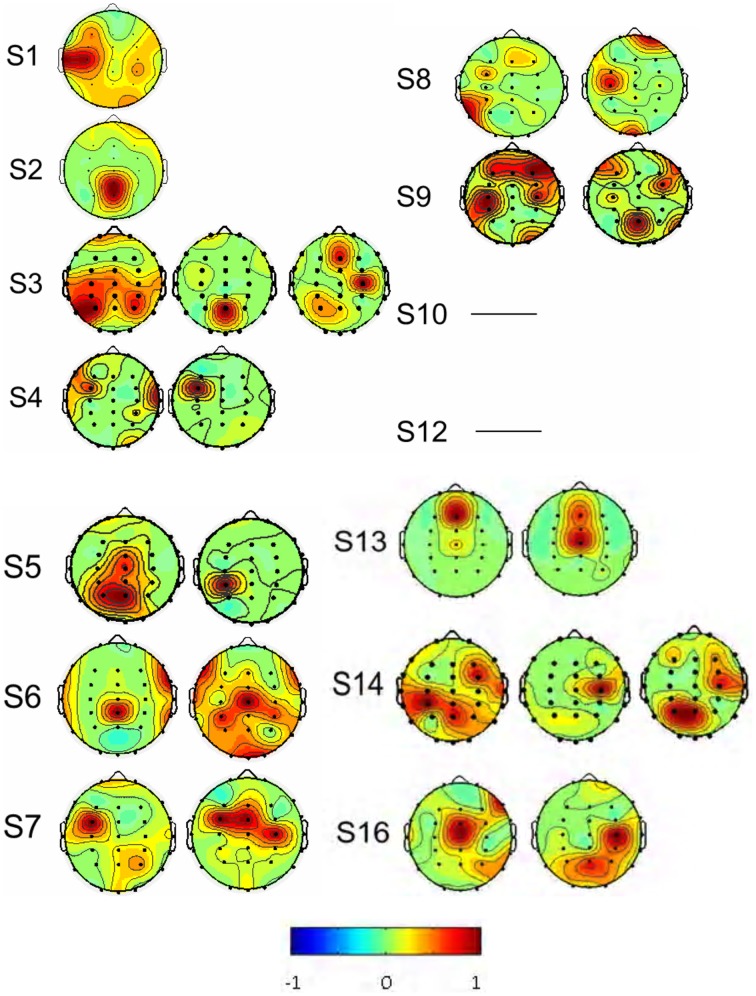
**The spatial distributions of the interested source signals from Fourier-ICA analysis**. Results were from 14 of the 16 subjects, and subjects S 11 and S 15 were excluded. No source components were found in the subjects S10 and S12. Signal space distribution was denoted by selected color circles and color intensity (–1 to 1) on the map. Blue represents –1, Red represents 1.

Based on the same data selection method used for subject S3 in Figures 5, 6, data from the rest of the subjects were examined to select the source components; and results are shown in Figure 7. F-ICA found interested ICs in 12 subjects (S1–S9, S13–S14, and S16) in the two same areas where ICs were found with T-ICA method. One IC area was distributed in the occipital region near Pz; similar to the traditional ICA. The other was distributed in the sensory-motor cortex region of C3 and C4. The ICs distributions of each patient in the Pz and C3/C4 regions are also depicted in Table 1; where the source signals are expressed as the sum of intensity distribution value of an IC in a region. F-ICA found ICs source signals in 12 of 14 subjects (86%) while T-ICA found signals in 10 of 14 subjects (71%); not a significant difference. However, the distributions of strong and weak source signals were very different between the two methods. Of the 12 subjects with ICs source signals found with F-ICA, 11 had strong source signals (intensity distribution value ≥1.0) located in primary motor cortex region (C3/C4) compared with only 5 with T-ICA (Tables 1, 2). Only one subject had weak source signals (intensity distribution value < 1.0) located in C3/C4 region with F-ICA compared with five with T-ICA (Table 1). The difference of strong and weak signal distribution in C3/C4 between T-ICA and F-ICA group was statistically significant (*p* = 0.029; Table 2). The distribution of strong source signals in the Pz area was similar between T-ICA and F-ICA (7 of 14 vs. 8 of 14) respectively.

**Table 2:** **Comparing the intensity distribution (ID) of source signals in C3/C4 between T-ICA and F-ICA group**.

ID^*^	**T-ICA**		**F-ICA**		**Total**	**χ2**	***p***
	***n***	**%**	***n***	**%**			
<1.0	5	50.00	1	8.33	6	4.774	0.029
≥1.0	5	50.00	11	91.67	16		
Total	10	100.00	12	100.00	22		

* Difference was statistically significant (p = 0.029) by chi-square test with comparing the intensity distribution (ID) of source signals between T-ICA and F-ICA group. ^*^ID value: < 1.0, weak source signals; ≥1.0, strong source signals. Data excluded subjects with ID value = 0.0 (Table 1).

### Comparing the number of source signals of F-ICA with that of T-ICA

The total number of interested source signals from the 12 subjects with F-ICA analysis was 768, twice that of 384 from T-ICA (*t* = –2.565, *p* = 0.010; Table 1). Next, we compared the source signals as expressed as intensity distribution values obtained from F-ICA with those from T-ICA at two different regions (Table 1). In the sensory-motor region C3/C4, the total values from the 14 subjects summed to 23.00 (average 1.64 ± 1.17) with F-ICA; more than the 10.5 (average 0.750 ± 0.620) obtained with T-ICA (*t* = –2.631, *p* = 0.021). In the Pz area, the total value from the 14 subjects with F-ICA summed to 12.50 (average 0.89 ± 0.53) which was still more than the 8.20 (average 0.59 ± 0.48) from T-ICA, but the difference was not statistically significant with *p* = 0.066 (*t* = –2.003).

## Discussions

The present study employed a hand-moving paradigm to investigate the electrophysiological correlates of action-related visual and auditory information processing with F-ICA. Independent components associated with 8–12 Hz activity were analyzed, which exhibited significant responses during task conditions and differences between the pre-learning and post-learning periods. These findings are consistent with the assumption that event-related desynchronization reflects activation patterns.

### The link of sound and movement through the visual learning

In this study, we used the sound of “moving Tower of Hanoi (TOH)” as a stimulus paradigm to connect the action-related sound with cognition through the visual learning. These phenomena may indicate that auditory and visual movement loop have a close relationship during the process of the auditory understanding. Our observation was in agreement with the results of previous studies showing that visual learning modulated comprehension of movement and auditory stimulation (Bangert and Altenmüller, 2003; Pineda, 2005; Li et al., 2011; Matyja, 2015).

Our results revealed that the subjects' response after pre-listening without seeing the movement was not significant or immediate. Previous studies also suggested that the sound of “moving TOH” can trigger action-related brain activity, but the sound alone cannot immediately produce the response (Gallese et al., 1996; Keysers et al., 2003). Others further reported that only after viewing the moving TOH animation, the subjects establish a connection between the sound and “TOH” images (Kohler et al., 2002; Le Bel et al., 2009). When hearing the same sound again, the subject would immediately associate the sound of TOH with the moving actions. The results from our F-ICA analysis further confirmed that the source signals were difference between pre-listening and post-listening, and that during the pre-learning phase sound was not associated with specific action. These smaller changes relative to the baseline in mu rhythm power spectrum could be regarded as event-related desynchronization/event-related synchronization (ERD/ERS) phenomenon. The changes relative to the baseline in both two phases reflected the activity of related neurons and the difference in the source signals. Hanoi-moving action related to auditory stimulation could cause activation of the motor cortex area of the brain. Thus, the function of auditory motor as a part of CDZs system may depend on the degree of integration between auditory and motor experience. Understanding this integration is of great significance for the study of cognition, language and hearing, especially in learning. Defects in neuronal activity of patients with diseases such as ASD may alter auditory motor response (Stewart et al., 2015). Currently, there was no feasible and effective method to diagnose those defects. The results from this study provide a potential method by monitoring mu rhythm ERD/ERS changes between “Listening Action” and related brain activity to detect auditory motor response. In the future, the minimum spatial change of source signals from F-ICA may detect the locations of defects in the brain that are responsible for listening recognition and mental retardation. It has been assumed that cerebral cortex has multiple regions that are associated with the sensory-motor cortex activation generated by auditory stimulation (Le Bel et al., 2009; Stewart et al., 2015).

### The algorithm and source signals analyzing

The objective of F-ICA analysis was to isolate source signals and find their corresponding spatial mode. In this study, two methods, the traditional T-ICA and F-ICA were compared using simulation data, and then adopted to analyze Hanoi experimental data. T-ICA tends to find the source of the largest non-Gaussian component of the signal and has often been used for single trial ERP (event-related potentials) extraction or artifact removal. The rhythmic EEG has a smaller non-Gaussian property, and usually is close to a Gaussian distribution. Consequently it was difficult to find the source independent components related to EEG rhythms using the traditional method, thus limiting the application of T-ICA in EEG rhythmic component extraction. In comparison, F-ICA can identify more and important ICs that were action-related, visual and auditory evoked EEG signals (Hyvärinen et al., 2010).

Although F-ICA has an advantage in EEG/EMG rhythmic activity extraction over T-ICA, it also faces some of the same problems as T-ICA. For example, in our study T-ICA did not find suitable source components in S4, S5, S9, and S10. Similarly, F-ICA failed to identify components in subjects S10 and S12. The reason was that both used the same source separation method and transformation matrix process. F-ICA extracted more effective independent components that are mixed with other signals according to Hanoi model, but the statistical analysis with real data showed that the model was strongly affected by the signals. Theoretically, such effects could be seen in the asymptotic variance of the estimators but might not be very relevant. Because most of the errors in practical analysis were due to violations of the model assumptions, such as independence and linearity, the effect of the violations was quite difficult to be analyzed.

Based on the results from our F-ICA analysis, two kinds of source signals were found, one in occipital top region and another in the motor cortex region. The meaningless sounds and actions related sounds can be established by learning. In the third phase experiment after the relationship was established, and when hearing the sound of moving Hanoi again, the subjects could recall just the video of the moving Hanoi, leading to the occipital parietal region activities associated with visual. Subsequently, they recalled the video with action meaning, leading to the activation of the motor cortex region. The component separation results of the traditional time-domain ICA showed that only one main source was located in the central partial occipital region which is not a pure motor region. It may involve part of the action region and part of the vision related region near Pz electrode of EEG. The results using F-ICA show that there are two main sources. One is near sensory-motor region C3 electrode, the other is near Pz electrode. These results suggested that in the traditional ICA method, the two source signals were integrated into one.

In 1924, German psychiatrist Berger invented EEG, and observed that the sound stimulation could inhibit α wave of the evoked EEG. In 1930, Wever and Bray used auditory nerve electrodes to detect cochlear nerve action potentials of cats and created the electrophysiological study of the auditory system (Hallpike and Rawdon, 1934). Then, K-complex wave, P300 wave, Auditory Evoked Potentials (AEP) and Brainstem Auditory Evoked Potentials (BAEPs) successively emerged (Campbell et al., 2005; Perlman et al., 2015; Tsai et al., 2015), and the reaction time of auditory evoked potential is shorter (the incubation period is usually 0–800 ms) in these studies. The visual cortex has two major pathways: parietal dorsal pathway and ventral pathway (Wang, 2006), and the auditory cortex information processing pathways has the dorsal object location and the ventral object recognition depends on double channel model. The auditory channels include: (1) primary auditory channel that is responsible for carrying the cochlear nerve information to reach the thalamus after 3~4 s relays, decoding and integrating the sound; (2) non-primary auditory channel that is responsible for the cochlear nerve information to reach each sensory-motor cortex after transmitting to the thalamus (Zhang et al., 2001). In the integrated dual auditory channel, the positive auditory channel converts the sound message to sensory-motor region (44 region and abdominal PMC) after auditory information decoding(Zhang et al., 2001), which is consistent with the F-ICA results in this study. In reverse auditory channel, stimulus associated with auditory attention and willingness at the Inferior Parietal Lobule (IPL) was showed to influence auditory neurons to select the information associated with various action regions (Wang et al., 2008).

### Auditory system and stimuli process

The Wernicke-Geschwind model explains that the auditory stimuli process was associated with actions. The auditory stimuli for the auditory cortex was processed by the auditory system into meaningful information in Wernicke's area, and transmitted to Broca's area by arcuate fasciculus. The auditory information was transformed into motion coding output which led to all kinds of movements (Rauschecker and Scott, 2009). Auditory system and stimulation task were closely related (Anderson et al., 1999). In this study, we developed experimental design to show that the auditory stimulus was associated with the action, and the source signals were found to be near Broca's area. Auditory-action recognition system relied on the action experience of the subject, thus the same sound can cause the motor cortex response. In F-ICA, in combination with the prior knowledge of time-domain, frequency-domain and airspace, one or more of the independent components corresponding to a particular component in EEG were identified. Thus, the interested components in the original signals associated with the task were extracted. Our results suggested that those interested components might be the cause of the visual and auditory stimuli evoked EEG response associated with movements. We used F-ICA method to analyze the spatial patterns of visual and auditory stimuli evoked EEG response associated with actions. We assume that the existing source signals should produce EEG response mapping to the scalp. Then, it is possible to get the spatial distribution of the source signal in brain sensory-motor cortex region or visual, auditory cortex region. It was of great significance that we identified the location of visual and auditory stimuli evoked normal human EEG response associated with actions. It was also an important step to reveal the association of the EEG signals processing results with the brain functions.

Action-related auditory stimulation (ARAS) could activate the brain area related to the action. The sound coupled with specific action content could serve as the stimulation. ARAS helped to find the neuronal activity in F5 area, sensory-motor area and Inferior Frontal Gyrus (IFG) of monkeys (Kohler et al., 2002; Keysers et al., 2003). When the tearing sound occurred, 13% of the neurons in the monkey's Primary Motor Cortex (M1) area were discharged. Once the monkeys heard the sounds related to the action of hand throwing sticks, peeling peanuts etc., the neurons in primary motor cortex area were also discharged, because the response was triggered from hearing the sound related to the same action. The action also activated the neuron discharge in sensory-motor area. The white noise and sound unrelated to the action, such as a monkey crying, could not activate neuron discharge in sensory-motor area (Kohler et al., 2002; Keysers et al., 2003). The human brain can also sense the sound related to the hand-related action-Hanoi moving as it was displayed in this research. We illustrated that the sounds related to the action of hand moving Hanoi could induce the change in the EEG signals distributed in the Primary Motor Cortex (M1) of the sensory-motor cortex.

## Conclusions

This study adopted Hanoi-moving video paradigm as an action-related visual and auditory stimulation to evoke EEG source signals, and used the F-ICA method to analyze the spatial distribution of the EEG signal sources. The results indicate that F-ICA is capable of distinguishing the two sources that were responses to action-related visual and auditory stimuli in normal healthy subjects. The C3 electrode near the sensory-motor region is associated with auditory stimuli and motor cortex activation with the movements and the electrode near Pz is associated with visual stimuli. The method used in this study could be applied to investigate the electrophysiological correlates of action-related visual and auditory information processing, and be potentially useful for the clinical diagnosis of conditions such as recognition dysfunction, language retardation and mental developmental disorders in the near future.

## Conflict of interest statement

The authors declare that the research was conducted in the absence of any commercial or financial relationships that could be construed as a potential conflict of interest.
